# Exploratory Study on Plasticiser Intake During Intermittent Fasting: Effects on Weight, Glycaemic Control and Vitamin D Levels in Type 2 Diabetes

**DOI:** 10.3390/toxics14050382

**Published:** 2026-04-29

**Authors:** Edwina Brennan, Priya Das, Pearl Wasif, Xianyu F. Wang, Jochen F. Mueller, Chang He, Jean V. Varghese, Alexandra E. Butler, Stephen L. Atkin, Naji Alamuddin

**Affiliations:** 1 School of Medicine, Royal College of Surgeons in Ireland-Medical University of Bahrain, Busaiteen 15503, Bahrain; ebrennan@rcsi.com (E.B.); pdas@rcsi.com (P.D.); psulaiman@rcsi.com (P.W.); satkin@rcsi.com (S.L.A.); naalamuddin@rcsi.com (N.A.); 2 Queensland Alliance for Environmental Health Sciences, The University of Queensland, 20 Cornwall St., Woolloongabba, Brisbane, QLD 4102, Australia; x.wang18@uq.edu.au (X.F.W.); j.mueller@uq.edu.au (J.F.M.); c.he@gdut.edu.cn (C.H.); 3 School of Environmental Science and Engineering, Guangdong University of Technology, Guangzhou 510006, China; 4 King Hamad University Hospital, Alsayh, Sheikh Eisa Bin Salman Bridge, Busaiteen 24343, Bahrain

**Keywords:** intermittent fasting (IF), type-2 diabetes (T2D), plasticisers, phthalate metabolites, vitamin D (VitD), glycaemic control

## Abstract

Introduction: Intermittent fasting (IF) is becoming increasingly popular as a method of weight management, but it is unknown whether it affects plasticiser intake with resultant changes in glycaemic control in diabetes and vitamin D (VitD) levels; therefore, this study was undertaken in a cohort of control and type-2 diabetic (T2D) subjects during Ramadan time-restricted feeding (TRF). Methods: In T2D subjects (*n* = 19) and controls (*n* = 31) undertaking TRF, 24 h urinary levels of phthalate metabolites, bisphenols and serum VitD were determined pre- and post-TRF by liquid chromatography–tandem mass spectrometry (LC-MS/MS). Anthropometric data and glycosylated haemoglobin (HbA1c) were measured. Results: T2D subjects were older (52 versus 36.73 years, *p* < 0.001), and had higher BMI (36.54 versus 27.67 kg/m^2^, *p* < 0.001), body weight (101.77 versus 80.36 kg, *p* < 0.001), and HbA1c (8.38 versus 5.46%, *p* < 0.001) compared to controls, while VitD levels did not differ (60.43 versus 63.95 nmol/L, *p* > 0.05). Post-TRF, HbA1c was unchanged in T2D subjects and there was no difference in weight, BMI or VitD. Increased mono-iso-butyl phthalate (MiBP) in T2D subjects (10 versus 6.1 ng/mL, *p* = 0.001) and mono-n-butyl phthalate (MnBP) in T2D subjects (37 versus 13 ng/mL, *p* = 0.018) and controls (8.3 versus 5.4 ng/mL, *p* = 0.007) were observed post-TRF; however, significance was lost after adjusting for baseline differences in age, BMI, and HbA1c using a general linear model (GLM) repeated-measures ANOVA. Despite having no median differences in DEHP (di-2-ethylhexyl phthalate) metabolites pre- and post-TRF, analyses revealed a significant time × HbA1c interaction for [mono(2-ethyl-5-carboxypentyl) phthalate, MECPP: *F*(1,42) = 4.79, *p* = 0.03, mono(2-ethyl-5-hydroxyhexyl) phthalate, MEHHP: *F*(1,42) = 8.56, *p* = 0.006, mono(2-ethylhexyl) phthalate, MEHP: *F*(1,42) = 4.64, *p* = 0.03 and mono(2-ethyl-5-oxohexyl) phthalate, MEOHP: *F*(1,42) = 8.19, *p* = 0.007] and time × group interactions [MEHHP: *F*(1,42) = 14.27, *p* < 0.001, MEHP: *F*(1,42) = 6.35, *p* = 0.01 and MEOHP: *F*(1,42) = 10.30, *p* = 0.003]. Estimated marginal means (adjusted for age, BMI, HbA1c, and VitD) further confirmed higher concentrations of DEHP metabolites [MECPP, MEHHP, MEHP, and MEOHP] in T2D participants over time compared with controls. Additionally, monomethyl phthalate (MMP) trajectories were significantly influenced by the time × group interaction (*F*(1,42) = 4.28, *p* = 0.04), with post-TRF elevations observed in T2D subjects. Vitamin D status was observed to modify mono(3-carboxypropyl) phthalate (MCPP) and MEP trajectories over time. Conclusion: Ramadan TRF is associated with changes in plasticiser metabolite levels, with estimated increased levels in T2D subjects versus healthy controls. Metabolite levels were influenced by HbA1c and vitamin D, though BMI was not observed to be a contributing factor.

## 1. Introduction

Weight management is key for improving glycaemic control in type 2 diabetes (T2D) through diet and pharmaceutical therapy [1]. Intermittent fasting (IF) is popular due to its contribution to enhanced weight loss, improved metabolic profiles and reduced risk of metabolic and cardiovascular disorders [2]. IF is associated with improved vitamin D (VitD) levels [3,4]. VitD deficiency is associated with obesity [5], and in those with T2D, supplementation is shown to improve glycaemic control [6,7], indicating the indirect benefits of IF on metabolism. However, benefits may be offset by ingesting chemical obesogens, including plasticisers, potentially impacting regulation of energy balance by altering lipid homeostasis to promote adipogenesis and lipid accumulation [8], thereby contributing to the obesity epidemic and associated metabolic disorders [9].

The most common regimens of IF are alternate day fasting (ADF), including modifications such as 5:2 fasting, and time-restricted feeding (TRF) [10]. TRF confines the consumption of calories to a specific time window of a day and differs from other forms of IF in that there are no restrictions on calorie intake, neither in type nor amount, during the eating window [10]. In Islam, the Ramadan TRF fast involves zero-calorie intake through eating or drinking from dawn to sunset and is strictly adhered to, with a reported 84% of Muslims with T2D observing the fast [11].

In plastic manufacturing, phthalates, derivatives of phthalic acid, provide flexibility while bisphenols increase rigidity. As phthalates are not chemically bound to the parent polymer, they have the potential to leach, resulting in exposure primarily through the ingestion of contaminated foodstuffs [12]. In a similar manner, Bisphenol A (BPA) migration occurs through diffusion of residual monomers and hydrolysis processes, with ingestion being the primary route of exposure [13]. While the migration rates of plasticisers from food packaging are dependent on several factors [13,14], studies report leaching at levels above regulatory allowed limits [15]. Although plasticisers have relatively short half-lives in the range of hours and are rapidly metabolised [16,17], they, their metabolites and analogues are associated with obesity and diabetes [12,18,19,20,21].

Plasticisers are thought to promote obesogenic effects through multiple pathways, including androgenic and anti-androgenic activity [22], disruption of the hypothalamic–pituitary–adrenal and/or –thyroid axes, and alterations in peroxisome proliferator-activated receptors (PPAR) signalling [23,24]. There is some evidence to support the theory that these plasticisers exert their effects through altered PPAR. For example, PPAR-γ, which plays a central role in regulating fat accumulation in adipose tissue by promoting adipogenesis and the control of insulin sensitivity, has been shown to be directly activated by benzyl butyl phthalate (BBP) [25] and the di(2-ethylhexyl) phthalate (DEHP) metabolite mono(2-ethylhexyl) phthalate (MEHP) [26]. In preadipocytes subjected to chronic low dose BPA exposure, a temporary rise in PPAR-γ expression was observed alongside enhanced lipid accumulation [27]. Similarly, BPS exposure resulted in upregulated PPAR-γ and induced lipid accumulation in preadipocytes [28].

Phthalate concentrations observed in Bahrain, though elevated, are consistent with findings from other Middle Eastern studies; however, one study indicates that exposure–response patterns vary across different plasticisers, with factors such as age and BMI influencing these relationships [29]. These variations may also be modified by TRF.

VitD (cholecalciferol) is synthesised in the skin following exposure to ultraviolet B (UV-B) radiation, which acts on 7-dehydrocholesterol. This precursor is subsequently hydroxylated in the liver at the 25-position to form 25-hydroxy VitD (25(OH)D_3_). In the kidneys, 25(OH)D_3_ undergoes further conversion by 1-alpha hydroxylase into its biologically active form, 1,25(OH)_2_D_3_ [30,31]. The active hormone exerts its effects by binding to the VitD receptor (VDR), forming a heterodimer with the retinoid X receptor [32]. In addition to this genomic pathway, more rapid responses can occur via membrane-associated VDR or through a membrane-bound steroid-binding protein, leading to activation of protein kinases A and C [33]. VitD levels may increase with TRF [4] and decrease with phthalate exposure [34,35,36].

Thus, the aim of this study was to compare the levels of the plasticisers (phthalate metabolites and bisphenols), before and after Ramadan TRF and to determine if there was a relationship with weight change, diabetes control (glycosylated haemoglobin A1c (HbA1c) levels) and VitD levels.

## 2. Methods

### 2.1. Study Design

This study adopted a prospective observational framework and was conducted at a single institution, the King Hamad University Hospital (KHUH), Bahrain. Ethical approval was granted by both KHUH (Ref: 21-406) and the Royal College of Surgeons in Ireland, Medical University of Bahrain (Ref: 210321). Participants were enrolled through convenience sampling via advertisements placed within the hospital and diabetes clinics. Individuals with T2D who volunteered were also invited to refer non-T2D relatives for participation.

Eligibility for participants with T2D was based on the World Health Organisation (WHO) diagnostic criteria for diabetes [37] and being on a consistent medication regimen for 3 months prior to study enrolment (diabetes treatment: metformin and an SGLT2 inhibitor). Eligibility for the non-diabetic control group required the absence of any diagnosed chronic disease. For both individuals with T2D and non-diabetic control participants, additional eligibility criteria included being older than 21 years of age and fasting for the whole month of Ramadan. All participants gave their written informed consent to take part in the study. The study consisted of two clinic visits encompassing a period of 11 weeks in 2021: an initial visit at 3 weeks before the month of Ramadan, and a final visit at 3 weeks after the month of Ramadan. For each visit, participants underwent routine non-fasting blood collection and anthropometric assessment. The 24 h urine samples were collected pre- and post-Ramadan, with all samples stored at −80 °C until analysis.

### 2.2. Anthropometric and Plasma Biochemical Measurement

Anthropometric measurements (height, weight, and waist circumference), blood pressure assessments and plasma biochemical assessments were carried out at baseline (3 weeks before the month of Ramadan) and at the final visit (3 weeks after the month of Ramadan). HbA1c levels were assessed using a haemoglobin testing system (VARIANT II TURBO Bio-Rad Laboratories, Inc., Hercules, CA, USA), in accordance with the manufacturer’s instructions. VitD concentrations were quantified by isotope-dilution liquid chromatography–tandem mass spectrometry (LC-MS/MS) (Waters, Milford, MA, USA) [38].

### 2.3. Plasticiser Measurement [29]

Urine sample preparation and plasticiser analysis: All glassware underwent thorough cleaning followed by heat treatment in a muffle furnace at 500 °C to eliminate potential plastic-derived contaminants. Items unsuitable for furnace treatment were sequentially rinsed using LC-grade n-hexane (1 wash), acetone (3 washes), gradient-grade methanol (3 washes), and HPLC-grade water (3 washes). Frozen urine samples were thawed prior to processing, and 50 µL aliquots were transferred into 2 mL amber vials. Each sample was spiked with 40 µL of carbon-labelled bisphenol and phthalate metabolite standard mixture (25 ppb in methanol). This was followed by the addition of 485 µL of ultrapure water and 25 µL of β-glucuronidase (*Escherichia coli*-K12; ≥3.5 units of specific activity). Samples were incubated at 37 °C for 90 min to allow enzymatic deconjugation. After incubation, 400 µL of 0.5% aqueous acetic acid (Merck, Darmstadt, Germany) was added and the mixture was centrifuged at 4800 rpm for 20 min prior to instrumental analysis.

Specific gravity: Urine-specific gravity (SG) was determined using a calibrated digital refractometer (UG-α; ATAGO Co., Ltd., Tokyo, Japan), with calibration performed using MilliQ water before each measurement. Specific gravity-corrected concentrations [SG-normalised concentration = crude concentration × (SGmedian − 1)/(SGsample − 1)] were used for all statistical analyses, where SGmedian refers to the median value of the specific gravity of the whole sample set and SGsample refers to the value of the specific gravity of the sample.

Instrument analysis: Analytes were quantified using a SCIEX Triple Quad™ 7500 liquid chromatograph coupled with a tandem mass spectrometer (LC-MS/MS) system—QTRAP^®^ Ready (SCIEX, Framingham, MA, USA). Chromatographic separation was performed using a Kinet x^®^ Biphenyl column (1.7 µm) (Phenomenex, Torrance, CA, USA) maintained at 45 °C. For phthalate metabolites, the mobile phases consisted of (A) an aqueous solution containing 1% MeOH (Merck, Darmstadt, Germany) and 0.1% acetic acid, and (B) an aqueous solution of 99% MeOH and 0.1% acetic acid. Mobile phases were prepared with MilliQ water. Bisphenol analysis employed comparable solvents without acetic acid. Detection was performed using scheduled multiple reaction monitoring (sMRM), with two ion transitions monitored per target analyte and corresponding internal standard. Instrument conditions and the results of quality assurance and quality control (QA/QC) for targeted biomarkers are included in the Supplementary Materials (Supplementary Tables S1 and S2, respectively).

As parent phthalate diesters are rapidly metabolised in the body, we examined thirteen metabolites as established biomarkers of phthalate exposure commonly used in human biomonitoring studies [35,39,40]. These include monoethyl phthalate (MEP) for diethyl phthalate (DEP) exposure; mono-n-butyl phthalate (MnBP) and mono-iso-butyl phthalate (MiBP) for di-n-butyl phthalate (DiBP); mono(2-ethylhexyl) phthalate (MEHP), mono(2-ethyl-5-carboxypentyl) phthalate (MECPP), mono(2-ethyl-5-hydroxyhexyl) phthalate (MEHHP) and mono(2-ethyl-5-oxohexyl) phthalate (MEOHP) for di-(2-ethylhexyl) phthalate (DEHP); monobenzyl phthalate (MBzP) for butyl benzyl phthalate (BBP); monomethyl phthalate (MMP) for dimethyl phthalate (DMP); mono-iso-nonyl phthalate (MiNP) for di-iso-nonyl phthalate (DINP); mono-n-octyl phthalate (MnOP) and mono(3-carboxypropyl) phthalate (MCPP) for di-n-octyl phthalate (DnOP); and monocyclohexyl phthalate (MCHP) for dicyclohexyl phthalate (DCHP). A further seven bisphenols were targeted, including bisphenol A (BPA), BP-AF, BP-AP, BPB, BPF, BPS, and BPZ. These bisphenols include legacy plasticisers such as BPA, in addition to structurally similar analogues that have been introduced as suggested safe alternatives.

### 2.4. Statistics

Demographic characteristics are presented pre- and post-TRF, separately, for T2D patients and healthy controls in terms of means and standard deviations for continuous data and frequencies with percentages for categorical data. Differences between T2D patients and healthy controls were examined using an independent *t*-test or Mann–Whitney U-test for continuous data, and chi-square tests for categorical data. Within-group differences were examined using a paired *t*-test or a Wilcoxon signed-rank test.

To minimise the impact of measurements beyond detection limits, values were estimated using multiple imputations, assuming a log-normal distribution as previously described [41]. Pre- and post-TRF plasticiser levels are reported as medians with interquartile ranges (IQRs). Differences in plasticiser levels between T2D patients and healthy controls were examined using Mann–Whitney U-tests, and within-group differences were examined using a Wilcoxon signed-rank test due to the non-normal distribution of the data. To adjust for significant baseline differences in age, BMI, and HbA1c between T2D and control groups, a general linear model (GLM) repeated-measures ANOVA was conducted, with baseline values of these covariates included in the model. Primary outcomes included the time main effect and time × group (T2D vs. control) interaction, testing differential plasticiser trajectories between T2D patients and the control groups. Secondary analyses examined time × covariate interactions (age, BMI, HbA1c, vitamin D). Estimated marginal means (EM means) with Sidak adjustment provided covariate-adjusted group comparisons at each time point. Both arithmetic means ± SD and medians [IQR] were reported to address skewness. Mauchly’s test assessed sphericity assumptions, with Greenhouse–Geisser correction applied if violated (all analyses reported with sphericity assumed, ε = 1.00). All analyses used two-tailed testing, with a significance threshold of *p* < 0.05. *p*-values for median plasticiser levels were adjusted for false discovery rate (FDR) using the Benjamini–Hochberg method. Analyses were carried out using SPSS version 31.0.1.0 (IBM SPSS, Armonk, NY, USA) and R software (2023.06.2-561).

## 3. Results

### 3.1. Demographics and Metabolic Parameters in T2D Patients and Healthy Controls Pre- and Post-TRF

**Figure 1:**
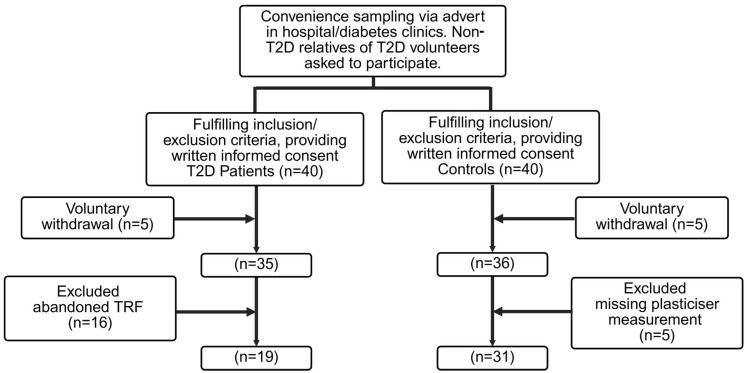
Participant recruitment flowchart.

A total of 40 T2D patients and 41 healthy controls were recruited in the study. In the T2D group, 5 subjects withdrew from the study and 16 T2D subjects were lost to follow-up (having stopped TRF during the study), and 19 subjects completed the study. In the control group, 5 withdrew, 5 were lost to follow-up and 31 completed the study (Figure 1). Baseline characteristics were broadly similar between completers and non-completers (Supplementary Table S3). The mean age was 52.0 ± 11.4 years among completers and 54.8 ± 10.7 years among non-completers. Measures of adiposity were also comparable, with mean BMI values of 36.5 ± 7.9 kg/m^2^ and 36.6 ± 9.0 kg/m^2^, respectively. Baseline glycaemic status was similar between the groups, with mean HbA1c values of 8.38 ± 1.12% in completers and 8.36 ± 1.36% in non-completers. Non-completers had a higher proportion of female participants than completers (52.4% vs. 30.0%).

T2D patients were significantly older (52 versus 36.73 years, *p* < 0.001), and had higher BMI (36.54 versus 27.67 kg/m^2^, *p* < 0.001), body weight (101.77 versus 80.36 kg, *p* < 0.001), and HbA1c values (8.38 versus 5.46%, *p* < 0.001) compared to healthy controls (Table 1). VitD levels between T2D patients and healthy controls did not differ (60.43 versus 63.95 nmol/L, *p* > 0.05). Gender did not differ between T2D patients and healthy controls (*p* > 0.05).

Following the Ramadan TRF, on average, T2D patients and healthy controls experienced weight losses of −0.88 kg and −0.37 kg, respectively, but weight loss was not significant and there was no change in BMI (Table 1). In addition, there was no difference in VitD levels in T2D patients and healthy controls. Healthy controls had a significant decrease in HbA1c values (5.46 versus 5.38, *p* = 0.012), while in T2D patients, HbA1c did not differ (Table 1).

### 3.2. Plasticiser Levels in T2D Patients and Healthy Controls Pre- and Post-TRF

Most measurements (94.2–100.0%) for four phthalate metabolites (MCHP, MiNP, MnOP, and MiDP) and five bisphenols (BP-AF, BPB, BPF, BPZ, BP-AP) fell below the detection threshold. Consequently, we restricted our analysis to the remaining ten phthalate metabolites (MBzP, MCPP, MECPP, MEHHP, MEHP, MEOHP, MEP, MiBP, MnBP, and MMP) and two bisphenols (BPA and BPS). MCPP levels were significantly higher in T2D patients compared to healthy controls prior to Ramadan TRF (1.5 versus 0.83 ng/mL, *p* = 0.04) (Table 2). The remaining eleven plasticisers had comparable levels in both groups. Post-TRF, plasticiser levels were comparable in T2D patients and healthy controls. MnBP levels were significantly increased in T2D patients and in healthy controls post-Ramadan TRF, at 37 versus 13 ng/mL (*p* = 0.018) and 8.3 versus 5.4 ng/mL (*p* = 0.007), respectively. MiBP levels were significantly increased in T2D patients (10 versus 6.1 ng/mL, *p* = 0.001) post-Ramadan TRF, but not healthy controls (Table 2). When FDR was applied, no significance remained.

### 3.3. Impact of Diabetes Status and Metabolic Covariates on Plasticiser Metabolite Changes

Unadjusted median analyses revealed group differences in MiBP and MnBP levels (Table 2), but covariate-adjusted GLM repeated-measures analysis showed no significant differences in MiBP and MnBP concentrations between T2D patients and controls (Table 3).

Medians (Table 2) and estimated marginal (EM) means (Table 4) confirmed stable MCPP levels across both T2D patients and controls. GLM repeated-measures analysis adjusted for baseline covariates (age, BMI, HbA1c and VitD) revealed that MCPP trajectories were significantly modified by age and VitD status (time × age *F*(1,42) = 6.55, *p* = 0.02; time × VitD (*F*(1,42) = 4.37, *p* = 0.04), but not by diabetes status (time × group *F*(1,42) = 0.52, *p* = 0.47), BMI, or HbA1c (Table 3). A significant time × VitD interaction was observed for MEP (*F*(1,42) = 4.65, *p* = 0.03) (Table 3), indicating that baseline VitD status modified MEP trajectories over time; there was no significant time*group interaction. MMP trajectories were significantly modified by the time*group interaction (*F*(1,42)= 4.28, *p*= 0.04) (Table 3), with higher EM levels in T2D patients post-TRF (Table 4).

Significant main effects of time were observed for MECPP [*F*(1,42) = 4.53, *p* = 0.03], MEHHP [*F*(1,42) = 10.9, *p* = 0.002] and MEOHP [*F*(1,42) = 6.86, *p* = 0.01]. Despite no median differences in MECPP, MEHHP, MEHP, and MEOHP pre- and post-TRF (Table 2), covariate-adjusted GLM repeated-measures analysis revealed significant time × HbA1c interactions [MECPP: *F*(1,42) = 4.79, *p* = 0.03, MEHHP: *F*(1,42) = 8.56, *p* = 0.006, MEHP: *F*(1,42) = 4.64, *p* = 0.03 and MEOHP: *F*(1,42) = 8.19, *p* = 0.007] and time × group interactions [MEHHP: *F*(1,42) = 14.27, *p* < 0.001, MEHP: *F*(1,42) = 6.35, *p* = 0.01 and MEOHP: *F*(1,42) = 10.30, *p* = 0.003] (Table 3). EM (adjusted for age, BMI, HbA1c, and vitamin D) confirmed higher concentrations of MECPP (marginal *p* = 0.05), MEHHP (*p* = 0.01), MEHP (*p* = 0.007) and MEOHP (*p* = 0.02) in T2D patients over time compared to controls (Table 4).

## 4. Discussion

This is the first study to examine plasticiser levels in the context of Ramadan TRF and their associations with changes in body weight (via BMI), VitD levels and diabetes control in a T2D cohort. In unadjusted models without FDR correction, we observed significantly increased median levels of MiBP in T2D patients, and MnBP in both T2D patients and in healthy controls at the end of Ramadan TRF. After adjustment for age, BMI, HbA1c and VitD levels, modelling of plasticiser levels over the TRF period identified a significant time × group and/or time × HbA1c interaction for MECPP, MEHHP, MEHP, MEOHP and MMP. VitD effects were observed for MCPP and MEP, with MCPP levels also influenced by age. Estimated levels of MECPP, MEHHP, MEHP, MEOHP and MMP following Ramadan TRF were higher in T2D patients compared to healthy controls. 

MiBP and MnBP are metabolites of DBP, a low-molecular-weight phthalate (LMWP) featuring ester side-chains of one to four carbon atoms in length [42]. DBP is widely used in PVC products, and evidence of its migration [43], as well as its detection in food and beverage products [44], has been reported. Although MiBP has been associated with a significantly higher risk of T2D [45], in this study, neither MiBP nor MnBP were associated with HbA1c levels or T2D status. While higher median levels of MiBP and MnBP were observed at the end of the TRF in unadjusted models, these differences were not maintained after FDR correction and covariate adjustment. These findings may reflect a relatively higher environmental exposure to DBP in this population, consistent with reports of elevated DBP exposure in the Middle East and increasing global trends [46]. The lack of association with metabolic parameters may be related to the short half-lives of MiBP and MnBP, reflecting recent exposure rather than cumulative tissue burden, as well as their weak ability to activate PPAR-α/γ signalling.

A recent meta-analysis indicates that in T2D patients prescribed oral hypoglycaemic agents, IF results in an HbA1c reduction of 0.54% [47]. In those with T2D following a similar treatment regimen to those in this study, Ramadan TRF was found to significantly reduce HbA1c [48]. In this study, glycaemic control (HbA1c) was significantly decreased in healthy controls over the TRF period, while there was no change in HbA1c in T2D patients. However, the magnitude of reduction in HbA1c in healthy controls was small (0.08%), and is unlikely to be clinically significant. The absence of change in T2D patients may be a result of underlying insulin resistance and medication use. These results suggest that Ramadan TRF in this cohort has a limited impact on glycaemic control in both healthy controls and individuals with T2D.

Our analysis revealed that DEHP metabolites (MECPP, MEHHP, MEHP and MEOHP) were influenced by either HbA1c or T2D, data that align with the Canadian Health Measures Survey [49], the male Korean National Environmental Health Survey [50], a study in an adult Chinese population [51] and the NHANES [52]. A similar pattern was observed for the DMP metabolite MMP. Additionally, we observed increased estimates of these DEHP metabolites following TRF in the T2D group compared to controls. Although not significant, MMP levels increased in T2D patients, while levels decreased in controls. DEHP is the most widely used high-molecular-weight phthalate (HMWP) [17] and is predominantly associated with dietary exposure, with levels of ≥300 μg/kg reported in food monitoring surveys [53], and levels as high as 2685 mg/kg reported in commercial food products [54]. DMP is a LMWP with reported migration of 0.6 mg/kg from polypropylene food containers [55]. While dietary intake is a major source of phthalate exposure, the group-specific effects point towards potential differences in metabolic handling. In the body, phthalates are metabolised via a two-phase process. Phase I involves rapid hydrolysis to primary metabolites by nonspecific esterases [56], followed by further oxidation catalysed by cytochrome P450 (CYP) enzymes to yield secondary metabolites. These metabolites subsequently undergo phase II conjugation, primarily glucuronidation mediated by UDP-glucuronosyltransferases (UGTs) in the liver [57]. Diabetes is associated with oxidative stress [58], which alters the activity of CYP enzymes [59]. The higher post-TRF concentrations of DEHP metabolites and MMP in this study following TRF in T2D patients may be reflective of impaired phthalate metabolite clearance through altered activity of CYP enzymes. Additionally, TRF may increase these lipophilic metabolite levels by promoting lipolysis and release from adipose stores, potentially overwhelming impaired hepatic clearance in T2D patients [60]. However, further studies are needed to distinguish between differences in exposure and differences in metabolism or clearance.

The whole cohort in this study exhibited mild–to-moderate deficiency in VitD levels (<75 nmol/L), a common occurrence among Arab populations, with 75% reported to have sub-optimal levels and 51% as VitD-deficient [61]. Baseline VitD levels were similar in T2D patients and controls and there were no differences in levels over the TRF period. The effect of Ramadan TRF on VitD levels is inconsistently reported, with some reports suggesting that there is no effect [62], as found here, and some suggesting that levels increase [4]. Interestingly, our modelling analysis revealed that MCPP and MEP metabolite levels were influenced by VitD. Few studies report on the relationship between VitD and phthalate levels, and although the findings are inconsistent, most report negative associations. In a US population, VitD was found to be negatively associated with the sum of DEHP metabolites [34,35], positively associated with MEP [35] and negatively associated with monocarboxyoctyl phthalate and monocarboxynonyl phthalate [35]. In a female-only cohort, VitD was negatively correlated with MCPP levels and with MiBP and MnBP in those with VitD deficiency [36], while in pregnant women, only negative associations with specific phthalate metabolites were observed [63]. VitD levels have also been reported to mitigate phthalate associations with all-cause mortality risk, as well as risks associated with cancer and cardiovascular disease mortality [64]. While the mechanisms explaining the associations between phthalate metabolites and VitD are not fully understood, phthalates are reported to interfere with CYP activity [65]. CYP enzymes catalyse the hydroxylation of VitD to its active form [30], while active VitD, through binding to VDR, regulates the expression of CYP enzymes [66] involved in phthalate metabolism. Additionally, phthalates have the potential to impact VitD via the disruption of VDR-mediated signalling [67]. At baseline, MCPP levels were significantly higher in T2D patients compared to healthy controls, and MEP had the highest median levels, as previously reported [29]. These findings are consistent with observations in T2D populations in Korea [68] and Saudi Arabia [69], as well as with MEP levels in the Middle East [46], suggesting that exposure levels in this cohort fall within previously reported ranges. However, overall median MCPP and MEP declines in T2D patients following the TRF period were non-significant, and although VitD levels remained stable across the intervention, this interaction indicates that pre-existing deficiency may impact MCPP and MEP trajectories. Further longitudinal studies with larger populations are warranted to examine the association between VitD and phthalates.

T2D patients had higher body weight at baseline compared to healthy controls; however, there was no difference in weight change between T2D patients and healthy controls over the TRF period, and changes in phthalates were not associated with BMI (which was included in the models as a measure of weight change over the TRF period). Studies report inconsistent associations between plasticiser exposure and weight change. In healthy women, after a 10-year follow-up from a one-time first-morning-void urine sample, MBzP, ƩDBP metabolites and BPA were associated with faster annual weight gain [70], while in post-menopausal women, MEP and MiBP levels were related to weight change over a 3-year period, but not over a 6-year period [71]. A recent review highlights conflicting data with regards to DEHP metabolite associations to BMI, with reports of positive, inverse and no associations, attributing inconsistencies to a number of factors including study population, sex, age, and race/ethnicity [58]. Additionally, most published studies employed single-sample urine collection, which may not accurately assess exposure given phthalate’s short half-life [9], unlike the procedure in this study, which used pooled 24 h collections.

The differing results for specific phthalate metabolites with weight change, HbA1c and VitD levels may be a result of racial differences, given reports that phthalate dose–response relations differ based on race and their disturbance on glucose homeostasis [72]. In addition, different populations have different plasticiser exposure profiles [20] and differ based on the prevalence of VitD deficiency [73]. Finally, individual populations vary regarding their genetic predisposition to T2D [74], which could be complicated by simultaneous exposure to plasticisers.

This study demonstrates strengths in the use of a uniform participant group, minimising variability related to ethnicity. In addition, it evaluates a broad range of plasticisers associated with endocrine and obesogenic effects. The inclusion of individuals with T2D, a population considered to be particularly susceptible, further enhances the study’s relevance. Moreover, the use of TRF represents a novel aspect that has not been previously explored. The increased accuracy of the phthalate determination using pooled 24 h collections, compared to single-sample measurements utilized in the literature, is a strength. In addition, most studies used creatinine correction, whereas in this study, we used specific gravity, which is preferable to creatinine adjustment [75], circumventing potential confounders like age, sex, gender and BMI [76]. The limitations of the study include the small sample size, and as this was an exploratory study, it may not have been adequately powered to detect changes in plasticiser levels over the TRF period, which in turn may have been too short a period to observe differences. While specific gravity correction mitigates the influence of urine dilution, it does not replace volume-based measurements of excretion; therefore, the results should be interpreted as relative changes in metabolite concentrations rather than absolute excretion rates. There was a loss of patients with diabetes through the study due to changes they reported in their diet midway through the study (due to stopping TRF), and therefore they were excluded. However, a descriptive comparison of baseline characteristics showed that completers and non-completers were generally comparable with respect to age, BMI, and HbA1c. Furthermore, the pharmacological effects of anti-diabetic therapies (SGLT2 inhibitors and metformin) in the T2D group were not considered in this study. The lowering of glomerular filtration rate (GFR) by SGLT2 inhibitors decreases the metabolic workload of renal tubules [77], and metformin contributes to renal protection by engaging AMP-activated protein kinases and promoting autophagy [78]. These renal and metabolic effects may alter plasticiser clearance, and therefore represent potential confounders. In addition, the study design did not allow for the identification of specific exposure sources. Behavioural and exposure-related factors, including dietary intake, the use of plastic food storage materials, physical activity and sleep patterns, were not assessed. Therefore, adjustment for these potential confounders was not possible. Such factors are likely to influence phthalate exposure and metabolism. Consequently, the observed associations should be interpreted with caution. Finally, our study focused on the novel impacts of plasticisers with weight change combined with strictly adhered-to Ramadan TRF, for which there are no comparisons in the literature. Future studies should take into account dietary (such as food frequency questionnaires and food storage practices) and behavioural proxies (such as physical activity and sleep questionnaires) to better characterise these relationships.

## 5. Conclusions

Ramadan TRF is associated with changes in plasticiser metabolite levels, with estimated increased levels in T2D patients versus healthy controls. Metabolite levels were influenced by HbA1c and vitamin D, though BMI was not observed to be a contributing factor. These findings suggest that both glycaemic control and VitD may play a role in modulating phthalate metabolite dynamics during Ramadan TRF; however, the underlying mechanisms, whether through exposure, metabolism or clearance, remain unclear. Further longitudinal studies incorporating dietary intake, food handling practices and specific exposure sources are warranted to confirm these associations.

## Figures and Tables

**Table 1:** Demographic and biochemical characteristics of T2D patients and healthy participants pre- and post-TRF.

		T2D (*n* = 19)		Healthy Controls (*n* = 31)		*p*-Value
Gender (%)						
Male		13 (68%)		20 (65%)		
Female		6 (32%)		11 (35%)		0.715
		Mean	SD	Mean	SD	
Age		52	11.49	36.73	9.65	**<0.001** ^a^
BMI (kg/m^2^)	Pre	36.54	7.95	27.67	5.19	**<0.001** ^a^
	Post	36.87	8.27	27.22	5.03	**<0.001** ^a^
	*p*-value	0.445 ^d^		0.232 ^c^		
HbA1c (%)	Pre	8.38	1.12	5.46	0.36	**<0.001** ^b^
	Post	8.18	1.23	5.38	0.37	**<0.001** ^b^
	*p*-value	0.383 ^c^		**0.012** ^c^		
Weight (kg)	Pre	101.77	17.40	80.36	17.11	**<0.001** ^b^
	Post	100.89	18.15	79.99	17.85	**<0.001** ^b^
	*p*-value	0.157 ^c^		0.341 ^c^		
VitD (nmol/L)	Pre	60.43	23.56	63.95	38.16	0.787 ^a^
	Post	62.74	27.24	60.49	29.35	0.757 ^a^
	*p*-value	0.411 ^c^		0.439 ^d^		

T2D, type 2 diabetes; BMI, body mass index; HbA1c, glycosylated haemoglobin A1c; VitD, vitamin D; statistical comparisons between T2D patients and controls: ^a^, Mann–Whitney U-Test; ^b^, independent-sample *t*-test. Statistical comparisons within-group pre- and post-TRF: ^c^, paired-sample *t*-test; ^d^, Wilcoxon signed-rank test. Statistically significant results (*p* < 0.05) are shown in bold.

**Table 2:** Median and interquartile-range plasticiser levels in T2D patients and healthy controls pre- and post-TRF.

		N < LOD	T2D (*n* = 19)		Healthy Controls (*n* = 31)			
Plasticiser (ng/mL)			Median	IQR	Median	IQR	*p*-Value ^a^	FDR Adj. *p*-Value
MBzP	Pre	-	0.37	0.33–0.83	0.23	0.13–0.5	0.054	0.32
	Post	-	0.36	0.18–0.75	0.35	0.22–0.44	0.667	0.76
	*p*-value ^b^		0.616		0.566			
MCPP	Pre	-	1.5	0.64–2.1	0.83	0.02–1.4	**0.04**	0.32
	Post	-	1.3	0.43–2.4	0.81	0.02–1.7	0.203	0.48
	*p*-value		0.647		0.381			
MECPP	Pre	-	15	5–27	6.2	3.2–19	0.114	0.34
	Post	-	8.8	4.3–24	8.8	3.1–19	0.646	0.76
	*p*-value ^b^		0.07		0.405			
MEHHP	Pre	1	4.8	1–18	1.1	0.57–11	0.093	0.34
	Post	5	7.5	0.76–24	1.7	0.45–7	0.110	0.48
	*p*-value ^b^		0.533		0.295			
MEHP	Pre	2	1.7	1.2–4.9	2.1	0.89–5.3	0.741	0.83
	Post	-	2.1	1.2–11	2.5	1.2–3.5	0.704	0.76
	*p*-value ^b^		0.122		0.65			
MEOHP	Pre	6	3.1	0.3–10	0.33	0.19–8.2	0.153	0.36
	Post	8	4.2	0.49–13	0.67	0.16–5.1	0.055	0.48
	*p*-value ^b^		0.26		1.000			
MEP	Pre	-	201	71–395	134	46–370	0.803	0.83
	Post	-	120	77–480	151	55–417	0.928	0.92
	*p*-value ^b^		0.338		0.593			
MiBP	Pre	8	6.1	0.57–17	2.5	0.89–20	0.542	0.72
	Post	6	10	2.6–37	5.8	1.5–16	0.187	0.48
	*p*-value ^b^		**0.001**		0.131			
MnBP	Pre	-	13	4–76	5.4	3–56	0.342	0.72
	Post	-	37	5.8–110	8.3	4–62	0.159	0.48
	*p*-value ^b^		**0.018**		**0.007**			
MMP	Pre	9	2.5	0.99–4.5	2.8	1.5–5.7	0.834	0.83
	Post	15	3.4	0.22–12	2	0.21–4.4	0.303	0.51
	*p*-value ^b^		0.198		0.166			
BPA	Pre	5	0.49	0.03–3.4	0.05	0.03–1.8	0.312	0.58
	Post	4	1.2	0.04–2.5	0.06	0.03–2	0.271	0.51
	*p*-value ^b^		0.586		0.745			
BPS	Pre	-	0.1	0.07–0.3	0.09	0.06–0.26	0.528	0.72
	Post	-	0.11	0.07–0.23	0.1	0.034–0.23	0.522	0.76
	*p*-value ^b^		1.000		0.871			

T2D, type 2 diabetes; MBzP, monobenzyl phthalate; MCPP, mono(3-carboxypropyl) phthalate; MECPP, mono(5-carboxy-2-ethylpentyl) phthalate; MEHHP, mono(2-ethyl-5-hydroxyhexyl) phthalate; MEHP, mono(2-ethylhexyl) phthalate; MEOHP, mono(2-ethyl-5-oxohexyl) phthalate; MEP, monoethylphthalate; MiBP, mono-isobutyl phthalate; MnBP, mono-n-butyl phthalate; MMP, monomethyl phthalate; BPA, bisphenol A; BPS, bisphenol S; IQR, interquartile range. Statistical comparisons between T2D patients and controls: ^a^, Mann–Whitney U-Test. Statistical comparisons within-group pre- and post-TRF: ^b^, Wilcoxon signed-rank test; limits of detection (LODs) (ng/mL) [MBbzP = 0.088; MCPP = 0.006; MECPP = 0.026; MEHHP = 0.11; MEHP = 0.44; MEOHP = 0.090; MEP = 0.26; MiBP = 0.50; MnBP = 0.55; MMP = 0.57; BPA = 0.02; BPS = 0.011]; false discovery rate (FDR) adj. *p*-value adjusted for multiple testing using the Benjamini–Hochberg method. Statistically significant results (*p* < 0.05) are shown in bold.

**Table 3:** Results of repeated-measures GLM for the plasticisers (time x covariate interactions).

Plasticizer	Effect	*F*(1,df)	*p*-Value	η_p^2^	Plasticiser	Effect	*F*(1,df)	*p*-Value	η_p^2^
MBzP	Time	0.0	0.97	0.00	MEP	Time	0.10	0.74	0.003
	Time × Age	1.76	0.19	0.040		Time × Age	0.008	0.93	0.00
	Time × BMI	1.10	0.30	0.026		Time × BMI	0.42	0.51	0.01
	Time × HbA1c	0.006	0.93	0.00		Time × HbA1c	0.08	0.77	0.002
	Time × VitD	0.02	0.88	0.001		Time × VitD	4.65	**0.03**	0.10
	Time × Group (T2D vs. Control)	0.70	0.79	0.002		Time × Group (T2D vs. Control)	0.004	0.95	0.00
MCPP	Time	0.17	0.67	0.004	MiBP	Time	0.06	0.79	0.002
	Time × Age	6.55	**0.02**	0.13		Time × Age	0.77	0.38	0.018
	Time × BMI	0.21	0.64	0.005		Time × BMI	0.008	0.92	0.00
	Time × HbA1c	0.85	0.36	0.02		Time × HbA1c	0.08	0.77	0.002
	Time × VitD	4.37	**0.04**	0.09		Time × VitD	0.96	0.33	0.02
	Time × Group (T2D vs. Control)	0.52	0.47	0.01		Time × Group (T2D vs. Control)	0.09	0.76	0.02
MECPP	Time	4.53	**0.03**	0.1	MnBP	Time	0.004	0.94	0.00
	Time × Age	0.11	0.73	0.003		Time × Age	0.04	0.83	0.001
	Time × BMI	0.08	0.77	0.002		Time × BMI	0.008	0.92	0.00
	Time × HbA1c	4.79	**0.03**	0.1		Time × HbA1c	0.003	0.95	0.00
	Time × VitD	0.74	0.39	0.02		Time × VitD	2.63	0.11	0.06
	Time × Group (T2D vs. Control)	3.68	0.06	0.08		Time × Group (T2D vs. Control)	0.009	0.92	0.00
MEHHP	Time	10.90	**0.002**	0.20	MMP	Time	2.61	0.11	0.06
	Time × Age	0.44	0.50	0.01		Time × Age	0.01	0.91	0.00
	Time × BMI	0.61	0.43	0.01		Time × BMI	0.88	0.35	0.02
	Time × HbA1c	8.56	**0.006**	0.17		Time × HbA1c	1.58	0.21	0.03
	Time × VitD	0.36	0.54	0.01		Time × VitD	0.14	0.70	0.003
	Time × Group (T2D vs. Control)	14.27	**<0.001**	0.25		Time × Group (T2D vs. Control)	4.28	**0.04**	0.1
MEHP	Time	2.27	0.13	0.05	BPA	Time	0.319	0.57	0.008
	Time × Age	0.42	0.51	0.01		Time × Age	0.015	0.903	0.00
	Time × BMI	1.65	0.20	0.03		Time × BMI	0.360	0.552	0.009
	Time × HbA1c	4.64	**0.03**	0.10		Time × HbA1c	0.008	0.931	0.00
	Time × VitD	1.27	0.26	0.03		Time × VitD	3.82	0.057	0.08
	Time × Group (T2D vs. Control)	6.35	**0.01**	0.13		Time × Group (T2D vs. Control)	1.86	0.669	0.004
MEOHP	Time	6.86	**0.01**	0.140	BPS	Time	0.12	0.72	0.003
	Time × Age	0.08	0.77	0.002		Time × Age	0.05	0.82	0.001
	Time × BMI	0.024	0.87	0.001		Time × BMI	0.05	0.82	0.01
	Time × HbA1c	8.19	**0.007**	0.16		Time × HbA1c	0.002	0.965	0.00
	Time × VitD	0.41	0.52	0.10		Time × VitD	2.60	0.114	0.06
	Time × Group (T2D vs. Control)	10.30	**0.003**	0.19		Time × Group (T2D vs. Control)	0.19	0.65	0.005

T2D, type 2 diabetes; BM1, body mass index; HbA1c, glycosylated haemoglobin A1c; VitD, vitamin D; MBzP, monobenzyl phthalate; MCPP, mono(3-carboxypropyl) phthalate; MECPP, mono(5-carboxy-2-ethylpentyl) phthalate; MEHHP, mono(2-ethyl-5-hydroxyhexyl) phthalate; MEHP, mono(2-ethylhexyl) phthalate; MEOHP, mono(2-ethyl-5-oxohexyl) phthalate; MEP, monoethylphthalate; MiBP, mono-isobutyl phthalate; MnBP, mono-n-butyl phthalate; MMP, monomethyl phthalate; BPA, bisphenol A; BPS, bisphenol S; df, degree of freedom; η_p^2^, partial eta-squared. Statistically significant results (*p* < 0.05) are shown in bold.

**Table 4:** Estimated marginal means and 95%CI of the plasticisers adjusted for baseline age, BMI, HbA1c and VitD.

		T2D	95% CI	Control	95% CI	*p*-Value
MBzP	Pre	0.73 ± 0.24	0.24–1.23	0.41 ± 0.17	0.06–0.76	0.40
	Post	0.72 ± 0.18	0.35–1.08	0.32 ± 0.12	0.06–0.57	0.16
MCPP	Pre	3.34 ± 1.88	−0.46–7.13	1.34 ± 1.32	−1.33–4.01	0.50
	Post	1.96 ± 1.71	−1.49–5.41	2.34 ± 1.20	−0.08–4.77	0.88
MECPP	Pre	13.42 ± 5.54	2.23–24.61	13.97 ± 3.89	6.11–21.83	0.15
	Post	30.77 ± 9.08	12.44–49.10	4.92 ± 6.38	−7.95–17.80	0.05
MEHHP	Pre	6.17 ± 5.16	−4.24–16.59	10.40 ± 3.62	3.07–17.72	0.60
	Post	33.06 ± 9.11	14.65–51.46	−4.41 ± 6.40	−17.07–8.78	**0.01**
MEHP	Pre	3.72 ± 2.79	−1.90–9.36	4.15 ± 1.96	0.19–8.11	0.12
	Post	11.06 ± 2.75	5.50–16.62	1.34 ± 1.93	−2.55–5.25	**0.007**
MEOHP	Pre	4.84 ± 3.16	−1.54–11.23	5.40 ± 2.22	0.92–9.89	0.90
	Post	18.14 ± 5.25	7.53–28.75	−1.03 ± 3.69	−8.48–6.42	**0.02**
MEP	Pre	237.08 ± 176.00	−118.11–592.28	325.87 ± 123.65	76.33–575.42	0.74
	Post	410.77 ± 541.49	−681.99–1503.5	540.19 ± 380.42	−227.5–1307.9	0.87
MiBP	Pre	12.36 ± 7.82	−3.42–28.15	12.32 ± 5.49	1.22–23.41	0.99
	Post	23.41 ± 15.18	−7.21–54.05	19.32 ± 10.66	−2.19–40.84	0.86
MnBP	Pre	61.03 ± 32.88	−5.33–127.40	29.92 ± 23.10	−16.70–76.54	0.24
	Post	78.42 ± 44.35	−11.08–167.92	44.72 ± 31.16	−18.15–107.60	0.33
MMP	Pre	2.80 ± 2.29	−1.82–7.44	4.79 ± 1.61	1.53–8.08	0.58
	Post	15.24 ± 5.59	3.94–26.54	−0.37 ± 3.93	−8.31–7.57	0.08
BPA	Pre	3.96 ± 0.98	1.71–5.67	−0.17 ± 0.69	−1.56–1.22	0.37
	Post	3.63 ± 1.69	0.21–7.06	0.75 ± 1.19	−1.65–3.15	0.96
BPS	Pre	1.00 ± 0.90	−0.82–2.82	0.17 ± 0.63	−1.10–1.46	0.56
	Post	1.04 ± 1.15	−1.28–3.37	0.70 ± 0.81	−0.93–2.34	0.85

T2D, type 2 diabetes; MBzP, monobenzyl phthalate; MCPP, mono(3-carboxypropyl) phthalate; MECPP, mono(5-carboxy-2-ethylpentyl) phthalate; MEHHP, mono(2-ethyl-5-hydroxyhexyl) phthalate; MEHP, mono(2-ethylhexyl) phthalate; MEOHP, mono(2-ethyl-5-oxohexyl) phthalate; MEP, monoethylphthalate; MiBP, mono-isobutyl phthalate; MnBP, mono-n-butyl phthalate; MMP, monomethyl phthalate; BPA, bisphenol A; BPS, bisphenol S. Statistically significant results (*p* < 0.05) are shown in bold.

## Data Availability

The data presented in this study are available on reasonable request from the corresponding author.

## Supplementary Materials

The following supporting information can be downloaded at https://www.mdpi.com/article/10.3390/toxics14050382/s1, Table S1: Instrument conditions; Supplemental Table S2: Concentrations (ng/mL urine) in blank samples, the limit of detection (LOD), and results of quality assurance and quality control (QA/QC) for targeted biomarkers; Table S3: Baseline characteristics of T2D participants.

## Abbreviations

IF, intermittent fasting; TRF, time-restricted feeding; BMI, body mass index; HbA1c, glycosylated haemoglobin A1c; DEHP, di-2-ethylhexylphthalate; LC, liquid chromatography; PPAR, perturbed peroxisome proliferator-activated receptors; HPLC, high-performance liquid chromatography; SG, specific gravity; LC-MS/MS, liquid chromatography–tandem mass spectrometer; sMRM, scheduled multiple reaction monitoring; SD, standard deviation; WHO, World Health Organisation; OGTT, oral glucose tolerance test; MEP, monoethyl phthalate; DEP, diethyl phthalate; MnBP, mono-n-butyl phthalate; MiBP, mono-iso-butyl phthalate; DiBP, di-n-butyl phthalate; MCPP, mono(3-carboxypropyl) phthalate; MEHP, mono(2-ethylhexyl) phthalate; MECPP, mono(2-ethyl-5-carboxypentyl) phthalate; MEHHP, mono(2-ethyl-5-hydroxyhexyl) phthalate; MEOHP, mono(2-ethyl-5-oxohexyl) phthalate; DEHP, di-(2-ethylhexyl) phthalate; MBzP. monobenzyl phthalate; BBP, butyl benzyl phthalate; MMP, monomethyl phthalate; DMP, dimethyl phthalate; MiNP, mono-iso-nonyl phthalate; DINP, di-iso-nonyl phthalate; MnOP, mono-n-octyl phthalate; DnOP, di-n-octyl phthalate; MCHP, monocyclohexyl phthalate; DCHP, dicyclohexyl phthalate; BPA, bisphenol A; BP-AF, bisphenol AF; BP-AP, bisphenol AP; BPB, bisphenol B; BPF, bisphenol F; BPS, bisphenol S; BPZ, bisphenol Z; LMWP, low-molecular-weight phthalate; HMWP, high-molecular-weight phthalate; VitD, vitamin D; CYP, cytochrome P450, UGT, UDP-glucuronosyltransferase, GFR, glomerular filtration rate
