# Supplementary material for: Exploratory Study on Plasticiser Intake During Intermittent Fasting: Effects on Weight, Glycaemic Control and Vitamin D Levels in Type 2 Diabetes
Source: Toxics. 2026 Apr 29;14(5):382. doi: 10.3390/toxics14050382 (PMC13211572; doi:10.3390/toxics14050382)
Supplement: Supplementary file 1 [file toxics-14-00382-s001.zip › toxics-4148812-supplementary.pdf]

Supplemental Table S1. Instrument conditions.

Compound	Abbr.	RT (min)	Transition 1	Transition 2	Internal standard	Transition 1
Monomethyl phthalate	MMP	4.11	179→77	179→107	$^{13}C_4$-MMP	183→79
Mono(3-carboxypropyl) phthalate	MCPP	4.47	251→103	251→165	$^{13}C_4$-MCPP	255→103
Monoethyl phthalate	MEP	4.78	193→77	193→121	$^{13}C_4$-MEP	197→79
Mono-iso-butyl phthalate	MiBP	6.84	221→77	221→134	$^{13}C_4$-MiBP	225→79
Mono-*n*-butyl phthalate	MnBP	7.04	221→77	221→71	$^{13}C_4$-MnBP	225→79
Mono(2-ethyl-5-hydroxyhexyl) phthalate	MEHHP	7.42	293→121	293→145	$^{13}C_4$-MEHHP	297→124
Mono(2-ethyl-5-carboxypentyl) phthalate	MECPP	7.55	307→159	307→113	$^{13}C_4$-MECPP	311→159
Mono(2-ethyl-5-oxohexyl) phthalate	MEOHP	7.70	291→121	291→143	$^{13}C_4$-MEOHP	295→124
Monobenzyl phthalate	MBzP	7.84	255→77	255→107	$^{13}C_4$-MBzP	259→107
Monocyclohexyl phthalate	MCHP	8.11	247→97	247→77	$^{13}C_4$-MCHP	251→97
Mono(2-ethylhexyl) phthalate	MEHP	9.52	277→134	277→77	$^{13}C_4$-MEHP	281→137
Mono-*n*-octyl phthalate	MnOP	9.68	277→127	277→77	$^{13}C_4$-MnOP	281→79
Mono-iso-nonyl phthalate	MiNP	9.70	291→77	291→139	$^{13}C_4$-MiNP	295→250
Bisphenol S	BPS	2.78	249→108	249→156	$^{13}C_{12}$-BPS	261→114
Bisphenol F	BPF	3.94	199→93	199→105	$^{13}C_{12}$-BPF	211→111
Bisphenol A	BPA	4.68	227→212	227→133	$^{13}C_{12}$-BPA	239→139
Bisphenol AF	BP-AF	4.89	335→265	335→177	$^{13}C_{12}$-BPAF	347→277
Bisphenol B	BPB	5.07	241→212	241→211	$^{13}C_{12}$-BPB	253→224
Bisphenol AP	BP-AP	5.54	289→274	289→273	$^{13}C_6$-BPAP	295→280
Bisphenol Z	BPZ	5.67	267→173	267→223	$^{13}C_{12}$-BPZ	279→179

Supplemental Table S2. Concentrations (ng/mL urine) in blank samples, the limit of detection (LOD), and results of quality assurance and quality control (QA/QC) for targeted biomarkers

**Compound**	**Abbr.**	**Concentration in blank samples (n=6; arithmetic mean)**	**Limit of detection (LOD)**	**Analytical accuracy**	**Analytical precision (relative standard deviation)**
Monomethyl phthalate	MMP	ND*	0.57	100%	5%
Mono(3-carboxypropyl) phthalate	MCPP	ND	0.0060	100%	6%
Monoethyl phthalate	MEP	0.18	0.26	100%	6%
Mono-iso-butyl phthalate	MiBP	0.25	0.50	94%	9%
Mono-*n*-butyl phthalate	MnBP	0.25	0.55	110%	7%
Mono(2-ethyl-5-hydroxyhexyl) phthalate	MEHHP	ND	0.11	100%	7%
Mono(2-ethyl-5-carboxypentyl) phthalate	MECPP	ND	0.026	98%	6%
Mono(2-ethyl-5-oxohexyl) phthalate	MEOHP	ND	0.090	97%	6%
Monobenzyl phthalate	MBzP	ND	0.088	97%	5%
Monocyclohexyl phthalate	MCHP	ND	0.11	91%	6%
Mono(2-ethylhexyl) phthalate	MEHP	0.23	0.44	110%	11%
Mono-*n*-octyl phthalate	MnOP	ND	0.18	92%	12%
Mono-iso-nonyl phthalate	MiNP	ND	0.080	89%	10%
Bisphenol S	BPS	0.0059	0.011	83%	3%
Bisphenol F	BPF	ND	0.66	91%	5%
Bisphenol A	BPA	ND	0.02	93%	5%
Bisphenol AF	BP-AF	ND	0.016	83%	5%
Bisphenol B	BPB	ND	0.030	93%	5%
Bisphenol AP	BP-AP	ND	0.033	94%	6%
Bisphenol Z	BPZ	ND	0.083	92%	5%

NB: *ND: not detectable

Synthetic urine was used as blank samples (n=6) and the limit of detection (LOD) for each analyte was defined as the mean of blank levels plus three times the standard deviation. If an analyte was not detected in the blank samples, its instrument detection limit (IDL) was used to calculate the LOD. LODs for the targeted analytes ranged from 0.0060 to 1.5 ng/mL urine.

Quality control (QC) samples (QCL, QCH) with a series of concentrations (0.5, 1, 2, 5, and 20 ng/mL urine; n=5 each and 25 in total) were prepared by fortifying the synthetic urine with target analytes. These QC samples were analysed together with the real urine samples. The analytical accuracy was assessed by dividing the measured concentrations by their expected concentrations stated above and expressing the results as percentages. The analytical precision was examined by averaging the relative standard deviation (RSD) of the results from each concentration level of the QC samples. All target analytes showed good analytical accuracy (83% – 110%) and analytical precision (RSD ≤ 12%). Detailed results are provided in Supplemental Table 2.

Supplemental Table S3. Baseline characteristics of T2D participants.

Characteristic	Completers (n = 19)	Non-completers (n = 21)
**Gender** (n, %)<br>Male<br>Female	 13 (68.0%)<br>6 (32.0%)	 10 (47.6%)<br>11 (52.4%)
**Age, years** (Mean ± SD)	52.0 ± 11.49	54.8 ± 10.7
**BMI, kg/m^2** (Mean ± SD)	36.54 ± 7.95	36.6 ± 9.0
**HbA1c, %** (Mean ± SD)	8.38 ± 1.12	8.36 ± 1.36*
**Weight, kg** (Mean ± SD)	101.77 ± 17.40	95.4 ± 24.2

T2D, type 2 diabetes; BM1, body mass index; HbA1c, glycosylated haemoglobin A1c. *HbA1c was available for 20 non-completers because one baseline HbA1c value was missing
